# Drug Abuse, Relapse, and Prevention Education in Malaysia: Perspective of University Students Through a Mixed Methods Approach

**DOI:** 10.3389/fpsyt.2015.00065

**Published:** 2015-05-06

**Authors:** Qiu Ting Chie, Cai Lian Tam, Gregory Bonn, Chee Piau Wong, Hoang Minh Dang, Rozainee Khairuddin

**Affiliations:** ^1^Jeffery Cheah School of Medicine and Health Sciences, Monash University Malaysia, Bandar Sunway, Malaysia; ^2^Graduate School of Education and Human Development, Nagoya University, Nagoya, Japan; ^3^Japan Society for the Promotion of Science, Tokyo, Japan; ^4^Centre for Research, Information and Service in Psychology (CRISP), Vietnam National University, Hanoi, Vietnam; ^5^Faculty of Social Science and Humanities, Psychology and Human Development, National University of Malaysia (UKM), Bangi, Malaysia

**Keywords:** drug abuse, contributory factors, drug knowledge, prevention education, higher education

## Abstract

In recent years, there have been increasing accounts of illegal substance abuse among university students and professional groups in Malaysia. This study looks at university students’ perceptions about this phenomenon. Students from Malaysian universities were asked for their impressions about drug availability and abuse, as well as factors contributing to drug abuse and relapse. The questionnaire also inquired into their knowledge and views regarding government versus private rehabilitation centers, as well as their exposure to, and views about, school-based drug-prevention education. Participants were 460 university students from five Malaysian states: Penang, Selangor, Kuala Lumpur, Sabah, and Sarawak. Results showed gender differences in perceptions of relapse prevention strategies, as well as factors leading to drug abuse and relapse. Students also believed that drug education would be more effective if initiated between the ages of 11 and 12 years, which is slightly older than the common age of first exposure, and provided suggestions for improving existing programs. Implications of student perceptions for the improvement of current interventions and educational programs are discussed.

## Introduction

Drug abuse continues to be a major problem in Malaysia (1), with almost half of the Malaysian prison population of 30,000 having been indicted for various drug-related offenses (2). Acknowledging the importance of drug abuse and relapse as a public health issue, the Malaysian government has implemented a number of programs aimed at curtailing drug distribution as well as improving drug-treatment programs. Starting from 2010, the National Anti-Drug Agency (NADA) undertook efforts to transform their existing rehabilitation programs to a so-called open-concept approach. Rather than focusing solely on compulsory rehabilitative treatment for convicted drug offenders (3), the open-concept approach provides drug users with the opportunity to receive treatment voluntarily at local community service centers without facing legal judgment or prosecution (4). This new approach was aimed at allowing relapsed users to seek treatment while continuing with their life and career (5), while also encouraging drug users with the motivation to change, to seek treatment voluntarily with no legal consequences.

Moving forward, to effectively deal with drug abuse, it will be important to get a clearer picture of the problem as it stands in the current social context. The transformation of Malaysian society to a more modern and globalized culture has caused much of the existing research on drug abuse to become out-dated. A case in point would be the change in drug users’ profiles in Malaysia over the years. Past research studies and national statistics showed the highest proportion of drug users among general laborers, followed by workers from service industries, agriculture, fisheries, and sales. The unemployed came in fifth place, in terms of occupational hierarchy among drug users (6–9). Drug users, in other words, were portrayed as from lower socioeconomic strata, often resorting to crime to fund their habit. Recent reports, however, suggest that drug users come from many different backgrounds, with white collar workers, civil servants, college students, and adolescents all represented in significant numbers (10). Findings from the longitudinal College Life Study (CLS) conducted in the University of Maryland found that there was widespread use of illegal substance with 23.6% of first-year students and 35.0% of third-year students meeting the criteria for substance use disorders (11). Working professionals with high-stress jobs and burnout such as social workers, doctors, and nurses are at equally, if not greater risk of engaging in drug use to relax or maintain punishing work schedules (12).

Such trends among Malaysian drug users warrant investigation. Global analytical reports from the years 2000 to 2008 suggest drug use starting at younger ages (pre-teens) in countries such as the United States, New Zealand, and Australia (13). Similarly, statistics based on admittance to rehabilitation programs in Malaysia from 2008 to 2012, suggest drug use being widespread among children aged 13–15 years and older (6). While statistics on drug use among Australians has consistently pointed to ages 18–24 as the years of most widespread use (14), similar statistics for Malaysia have fluctuated since 2008 (6). In 2008 and 2009, admissions to rehab were highest for users aged 25–29 years. By contrast, the years 2010 and 2011 saw 19- to 24-year olds being admitted at the highest rate, while in 2012 and 2013 those aged 30–34 years were most heavily represented (6, 7). It is not clear what the root of these changes is, but it does appear either that the pattern of drug use in Malaysia are shifting or that, since the legal changes in rehab availability, many users from different walks of life are seeking help who might not have done so in the past. The latest available statistics on drug types, which were commonly misused by Malaysian drug users in 2013, cited opiates (75.07%) as the highest ranked substance, followed by methamphetamines (13.58%), cannabis (8.82%), amphetamine-type substances (ATS) pills (2.23%), and psychoactive or psychopharmaceutical pills (0.22%), although it was not known which substance types were more highly used among drug users of different age groups (7).

The possibility that people from many different, non-criminal backgrounds are engaging in drug use is also illustrated by recent research showing individual differences in the ability to use drugs without developing dependence (15). Ersche et al. (16), for example, examined the personality traits and neural correlates associated with stimulant dependence. They compared 27 individuals who used cocaine recreationally for a minimum period of 2 years without exhibiting substance-dependent behavior patterns to 50 individuals with stimulant dependence. Non-dependent, recreational users were able to engage in drug use in social situations without affecting their daily functioning (i.e., relationships with family and friends, school and work responsibilities) possibly because they showed lower overall levels of compulsivity and impulsivity than those who were dependent, even while demonstrating similar levels of sensation-seeking (16). Understanding drug use and abuse, thus, is probably more complicated than previous Malaysian statistics suggested, with a much greater number of casual or recreational users among the educated and higher social strata than was previously assumed.

### Current study

This study thus aimed to broaden our understanding of the issues by taking a different approach. Rather than just looking at users or those who have been admitted to drug treatment programs, we chose to look at the views and opinions of the general public and non-users about the availability and use of illicit substances in Malaysia. Although previous research involving non-user populations has looked at reasons for non-use of illicit drugs (17) as well as perceptions of risk in Latin American countries (18), this kind of research has not been conducted in Malaysia. Understanding the mind-set of the non-user population, their conceptions and misconceptions about drug use, as well as related attitudes is, we feel, important for evaluating the effectiveness of existing drug education programs as well as for designing government health programs (19).

A second goal of this study was to understand the impression among university students, who are generally understood to be at high risk for drug use (20), of existing educational programs. The Malaysian government has initiated two programs, “SHIELDS” and “Tomorrow’s Leader,” which are aimed at providing drug education and prevention in public and private universities (7). However, there is little information available on the effectiveness of these and other programs conducted at various universities. Thus, this study also obtained qualitative feedback from students on their past experiences with drug education and prevention programs.

The research questions formulated in this study are:

(a) What are students’ perceptions of the types of drugs are commonly used and easily accessible?
(b) What are students’ perceptions about the contributory factors of drug abuse and relapse? Are there gender differences in this perception?
(c) What are students’ perceptions about effective strategies for preventing drug relapse? Do gender differences exist in this perception?
(d) What are the preferred media for sharing information and learning about drug abuse issues?
(e) How much awareness do students have about drug rehabilitation programs and what is their perception of the relative effectiveness of government and private rehab centers?
(f) Do the students have past exposure to school-based prevention education? What is their perception of the appropriate timing for prevention education?
(g) What are the students’ suggestions for improving drug education?

## Materials and Methods

### Ethics

The design and procedure of this mixed methods study were reviewed and approved by the Monash University Human Research Ethics Committee (MUHREC: CF13/511 – 2013000227).

### Recruitment and participating criteria

Purposive sampling was used to recruit participants for this study. An online advertisement to recruit participants was posted on university websites and social networking sites. The advertisement contained a brief summary of the research and the web link for the questionnaire. The criteria for participating were: participants should not have prior experience of illegal substance use and should be above 18 years. A wider age criterion was set to obtain participants within the at-risk age group but not excluding mature students. Mature students in this sample consist of individuals who were studying while juggling professional careers.

### Material

The online-administered Student Perception Questionnaire (SPQ) consisted of 26 items and was designed with a mixture of close-ended and open-ended questions. The SPQ was mostly self-developed although two scales used in this questionnaire: (a) Causes of Drug Misuse Scale (CADAS) and (b) Cures for Drug Misuse Scale were adapted from Cirakoglu and Isin (21). The questionnaire begins with basic demographic items to obtain basic background information such as gender, age, ethnicity, religion, and educational status. This was followed by the CADAS and CUDAS, which were rated on 5-point Likert scales. Other items were presented as ranking items, yes/no response, checklists, contingency questions, and open-ended responses.

Items in the SPQ cover seven research scopes: (a) the types of drugs commonly abused and easily available, (b) reasons for drug abuse and relapse, (c) strategies to overcome drug addiction and relapse, (d) prior knowledge about drug rehabilitation programs and treatment effectiveness, (e) information resource on drug abuse, (f) age appropriate for drug-prevention education, and (g) suggestions for improving drug education. The online questionnaire can be accessed through the following link: http://tinyurl.com/l6bgxkq. It takes approximately 30 min to complete the SPQ in a single sitting.

### Procedure

Students who were interested in participating clicked on the web link provided. Subsequently, an in-depth explanatory statement detailing the purpose, procedure, risks, benefits, and contact details of the researcher was provided. Information on professional help services was also included for students who experienced any form of discomfort or required assistance. After students indicated their consent to participate, the questionnaire items appeared in their browser window. Participation was voluntary and anonymous. Students who wished to withdraw from the study could do so without consequences by simply closing their web browser.

### Pilot study

A pilot study involving 50 students from Monash University Malaysia was conducted to test the reliability of the five constructs developed. Four out of five constructs were within acceptable to good reliability range (0.6 ≤ α < 0.9). Only one construct evaluating students’ knowledge and perception about drug rehab programs had poor internal consistency (α = 0.22). Further examination of this construct brought to the conclusion that these items could not be removed, as most questions were contingency response items. The overall internal consistency of the SPQ (α = 0.69) was found to be within acceptable levels of reliability (0.6 ≤ α < 0.7). In addition, the content validity of the questionnaire was performed by a panel of two experts who have done extensive work using the qualitative or mixed methods research approach and were previously engaged in research projects related to drug misuse and relapse. Both experts evaluated the clarity and representativeness of the questions. Several items on drug rehab programs were rephrased and open-ended questions about the appropriate medium for sharing information on drug misuse and prevention education were extended.

### Participants

Within a period of 1 year (June 2013–June 2014), 500 students from colleges and universities within the Malaysian states of Selangor, Penang, Kuala Lumpur, Sabah, and Sarawak responded to the advertisement and questionnaire. Of the respondents, 40 were excluded because they did not completely fill out the questionnaire. Thus, data from 460 students were included in the analysis. The age range was from 18 to 56 years and the mean age was 21.60 (SD = 3.547). There were more female participants (74.3%) compared to males (25.7%), in part, due to the higher female gender composition of students in higher learning institutions in Malaysia compared to males. There were 446 Malaysians (97.0%) and 14 participants (3.0%) of different nationalities. Most of the participants were from undergraduate Bachelor degree programs (74.3%) whilst 12.0% had a Diploma or equivalent; 6.5% of participants were at the Postgraduate level (Master/PhD), while 7.2% of participants reported being enrolled in pre-university or foundation programs.

## Results

### Drug types: Commonly misused and easily accessible

Students were asked which drugs were most frequently or commonly used as well as which were most accessible or available. Ten drug types were ranked for commonality (1 = most commonly used to 10 = least commonly used) and availability (1 = most easily available to 10 = least easily available). To determine the highest proportion of rankings for each drug type, mode values were obtained as shown in Table 1.

**Table 1:** **Commonality and ease of availability of substances according to mode rankings**.

Type of drugs	Ranking mode (*N* = 460)	
	Commonality	Availability
Ecstasy	1	1
Heroin	1	2
Morphine	5	5
Kratom/ketum leaves	10	1
Cannabis	1	1
Opiates	7	7
Methamphetamine	3	6
Amphetamine	9	8
Psychoactive pills	8	10
Ketamine	10	10

Ecstasy and cannabis were most often rated as 1, indicating that they were perceived as the two substances, which were most commonly used and easily available. Heroin was also ranked as most commonly used and second most easily available substance. Ketum leaves, a type of herbal drug that is more customary in Southeast Asian countries, was also ranked as most easily available but least commonly used. Methamphetamines were ranked third in terms of commonality but only obtained a rank of 6 in availability. Morphine was mid-rank in both commonality and availability, with a score of 5. Opiates received a rank of 7 in commonality and availability, while psychoactive pills and ketamine were perceived as the most difficult substances to obtain with a ranking of 10. Ketamine was also ranked as the least commonly used substance, together with ketum leaves.

### Contributory factors of drug abuse and relapse

The students rated the extent to which they agree or disagree with the given statements on reasons for drugs abuse, on a 5-point Likertscale. The Mann–Whitney *U* analysis was selected to analyze gender differences as the data were not normally distributed when a Shapiro–Wilk test was conducted (*p* < 0.05). The Mann–Whitney examines the differences in ranked positions and scores are ranked from the lowest to the highest. The highest mean rank indicates that there are a greater number of high scores within it and vice versa. The analysis on drug use factors as shown in Table 2 revealed significant gender differences for “experiencing unemployment” [*U*(459) = 17237.00, *z* = −2.45, *p* < 0.05]. Based on mean rankings, more female students rated significantly higher scores for unemployment as a reason for drug use in comparison to male students. There were two factors that male students consistently ranked higher than female students, which was “not educated” [*U*(459) = 18300.50, *z* = −1.56, *p* > 0.05] and “is weak-willed” [*U*(459) = 19840.50, *z* = −0.28, *p* > 0.05] although this difference was not significant.

**Table 2:** **Gender differences in the perception of contributory factors**.

Drug use factors	Participants (*N* = 460)		Mann–Whitney *U*		
	Male (*n* = 118)	Female (*n* = 342)	
				
	Mean rank	Mean rank	*U*	*z*	*p*
To rid of personal sufferings	212.97	236.55	18110.00	−1.83	0.07
Problematic family communication	218.70	234.57	18786.00	−1.20	0.23
Life stresses	221.27	233.68	9089.00	−0.96	0.34
Curiosity	217.78	234.89	18676.50	−1.27	0.20
Uneducated	246.41	225.01	18300.50	−1.56	0.12
Influence of media portrayals	223.58	232.89	19361.50	−0.69	0.49
Drug-dependent peers	215.79	235.57	18442.50	−1.50	0.13
Social environment	226.27	231.96	19679.00	−0.44	0.66
Weak-willed	233.36	229.51	19840.50	−0.28	0.77
Unemployment	205.58	239.10	17237.00	−2.45	<0.05

Students were given the option to select as many reasons as they felt applicable. Based on the number of “yes” responses, lack of family support (*n* = 397) was cited as the major reason for relapse, followed by lack of self-efficacy (*n* = 377) and peer influence (*n* = 357) as can be seen in Table 3. A chi-square test was also run to determine gender differences in ratings of relapse factors. The results demonstrate that there were no significant gender differences in ratings of relapse factors.

**Table 3:** **Gender comparisons of contributory factors of drug relapse**.

Drug relapse factors	Participants (*N* = 460)				Pearson’s chi-square test	
	Male (*n* = 118)		Female (*n* = 342)	
	No	Yes	No	Yes	χ^2^ (df = 1)	*P*
Lack of self-efficacy	20 (16.9%)	98 (83.1%)	63 (18.4%)	279 (81.6%)	0.129	0.782
Lack of family support	21 (17.8%)	97 (82.2%)	42 (12.3%)	300 (87.7%)	2.258	0.161
Lack of community support	45 (38.1%)	73 (61.9%)	105 (30.7%)	237 (69.3%)	2.206	0.141
Lack of employer support	78 (66.1%)	40 (33.9%)	229 (67.0%)	113 (33.0%)	0.029	0.475
Peer influence	28 (23.7%)	90 (76.3%)	75 (21.9%)	267 (78.1%)	0.163	0.387

### Effective strategies: Drug relapse

Students were required to rate the extent in which they agree or disagree with statements on strategies in overcoming drug relapse according to a 5-point Likert scale. Analysis from the Mann–Whitney *U* test shows gender differences for four strategies (see Table 4). More females rated significantly higher scores for “building supportive social networks” [*U*(459) = 16570.00, *z* = −3.18, *p* < 0.01], “learning stress management” [*U*(459) = 16736.50, *z* = −3.02, *p* < 0.01], “maintaining constant communication with recovery doctors” [*U*(459) = 17359.00, *z* = −2.45, *p* < 0.05], and “consulting rehabilitation centers” [*U*(459) = 17249.00, *z* = −2.57, *p* < 0.05] as strategies to overcome drug relapse in comparison to males.

**Table 4:** **Gender differences in the perception of strategies to overcome drug relapse**.

Relapse strategies	Participants (*N* = 460)		Mann–Whitney *U*		
	Male (*N* = 118)	Female (*N* = 342)	
				
	Mean rank	Mean rank	*U*	*z*	*p*
Forget the past and make life changes	232.75	229.72	19912.00	−0.23	0.82
Breaking unhealthy relationships	228.96	231.03	19996.50	−0.16	0.87
Keeping busy with healthy activities	219.93	234.15	18931.00	−1.14	0.26
Listen to music	215.09	235.82	8359.50	−1.53	0.13
Building supportive social networks	199.92	241.05	16570.00	−3.18	<0.01
Caution with medication	212.37	236.76	18038.50	−1.87	0.06
Learning stress management	201.33	240.56	16736.50	−3.02	<0.01
Constant communication with recovery doctors	206.61	238.09	17359.00	−2.45	<0.05
Consult rehab centers	205.68	239.06	17249.00	−2.57	<0.05
Limit places to visit	217.16	235.10	18604.00	−1.31	0.19
Do not carry too much money	224.74	232.49	19498.50	−0.57	0.57
Believe in overcoming problems	215.91	234.82	18358.00	−1.44	0.15
Be active in skillful areas	226.17	232.00	19666.50	−0.45	0.65

### Preferred information medium

A higher proportion of students seek information about drug abuse issues on their own initiative (*n* = 267, 58.0%) whereas 192 (41.7%) students did not.

#### Medium for Learning

Subsequently, the students had to rank the resources used to research about drug abuse and prevention on a 5-point scale (1 = most preferred resources to 5 = least preferred resource). Mode values were obtained to determine the highest proportion of rankings for each resource (see Table 5). Internet websites and blogs were ranked in first place by 41.7% of respondents as the most preferred information medium for such issues. This was followed by newspaper and magazine articles (20.9%), which received a mid-range rank of 3 as well as brochures, pamphlets, or posters (15.9%) received a ranking of 4. Both social sites (24.8%) and books (18.9%) were rated as the least preferred resource with a ranking of 5, respectively.

**Table 5:** **Information resource on drug misuse and prevention issues by ranking mode**.

Resources	Ranking mode	Responses (%)
Internet resources (websites, blogs)	1	192 (41.7)
Newspaper/magazine articles	3	96 (20.9)
Brochures/posters/pamphlets	4	73 (15.9)
Social sites (Facebook/Twitter)	5	114 (24.8)
Books	5	87 (18.9)

#### Medium for Sharing

**Figure 1:**
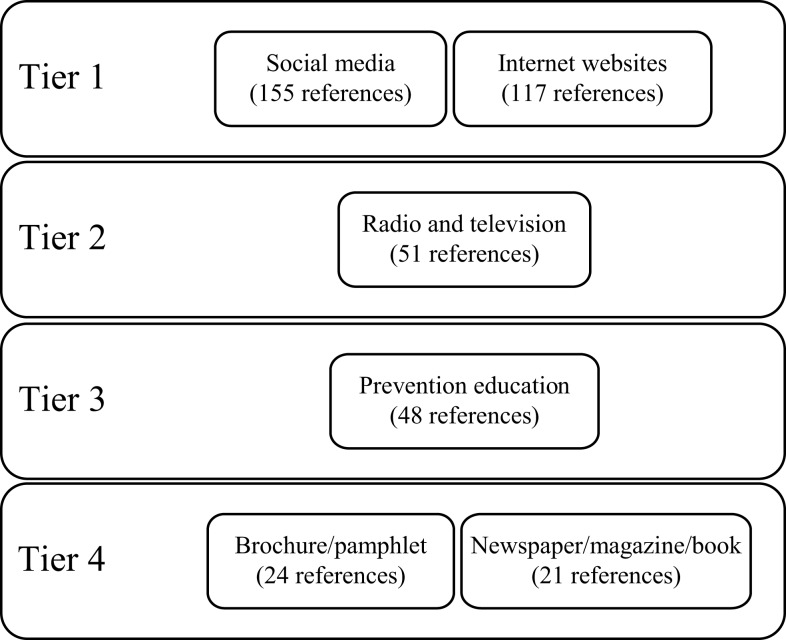
**The four most highly cited medium for information-sharing**.

The students were encouraged to provide opinions on what would be the best medium to share information. A tier system to rank the medium was developed based on range of citations by students. The classification of the tier system are as follows: (a) Tier 1: medium, which received the highest range of references (i.e., >100), (b) Tier 2: medium with the second highest reference range (i.e., >50), (c) Tier 3: medium with the third highest reference range (i.e., >40), and (d) Tier 4: medium with the fourth highest reference range (i.e., >20). As shown in Figure 1, social media and internet websites were the most highly cited resource for sharing information (Tier 1). The ease of sharing information to a wider audience and the wide accessibility of social network accounts were among the reasons cited by students for using social media. Many students reported that their knowledge had been greatly improved with social media sites. Informational websites and group forums were also popular among students who cited the ease of searching for information and perceived anonymity as reasons for using these resources.

‘… most of the people are addicted to social networking, or even they are not addicted to it, they will still glance through information on social network. People tend to share information around and this would be a great time for them to share out information about drug abuse.’ [US004]

‘… sharing it through internet resources like websites and blogs. This is because nowadays people used to make a research by doing online and the internet is the easiest way to access the drug matters.’ [US152]

Radio and television were the second most cited source of information among students.

‘Infomercials on television and radio would be a good way to inform the public about drug abuse or drug prevention strategies.’ [US007]

‘A documentary is always interesting, make like an expose about drugs and the effects, it’s fascinating to watch. Make it serious but in a comedic way because it might make people remember the info better.’ [US032]

Prevention education was the third most commonly cited source of information. The students acknowledged that prevention programs are important for providing early awareness of and exposure to the dangers of drug abuse. According to some students, many do not have the desire to read, learn, or investigate on their own. Thus, they felt that conducting compulsory programs would be beneficial.

‘Create awareness early in schools before the youths can actually get access to the drugs.’ [US035]

‘… through educational organisations. It is rather unlikely that a heavy drug abuser would take the initiative to search for drug related abuse information on the internet. Plus a person involved in such activities would be more likely than others to have friends that also abuse drugs.’ [US168]

The fourth most commonly cited source of information (Tier 4) consisted of printed materials such as brochures, pamphlets, and posters, as well as newspaper, books, and magazines. Brochures, pamphlets, and posters were cited as easy to read, with important facts briefly summarized and information presented creatively. Students suggested that brochures, pamphlets, and posters be given out in conjunction with drug-prevention seminars, to reinforce the knowledge gained. Some also suggested having committed columns in popular newspapers and magazines dedicated to substance abuse and other related mental health topics as a way of increasing awareness among the public. Newspaper and magazine articles by field experts and established columnists were seen as more influential and their messages easily acceptable to the public. However, the students highlighted a need for more books on psychiatry and substance abuse in bookstores and local libraries.

‘Brochures and pamphlets because it is usually brief and easy to read. It contains the important facts and it’s easy to share or pass around.’ [US014]

‘Greater availability of books on psychiatry and substance abuse is needed…’ [US114]

‘… newspaper or magazine articles would be effective mediums to share information about drug abuse or drug prevention strategies because these two mediums are very easily and widely accessible to the mass public.these two mediums are also known to have a large influential impact on society, thus the message or information is deemed more easily acceptable to the public.’ [US160]

### Knowledge: Rehabilitation and its effectiveness

A major proportion of students (*n* = 293, 63.7%) reported having knowledge about drug rehab programs as compared to 167 (36.3%) students who did not. A slightly higher proportion of students (45.9%) believed that there is a difference in treatment effectiveness between government and private rehab centers as compared to 42.0% who believe that treatment effectiveness was equal. Fifty-six students (12.2%) chose not to answer this question.

Findings on the effectiveness of rehabilitation centers demonstrated that a higher proportion of students (48.9%) viewed private centers as more effective than government rehab centers (9.8%). One respondent (0.2%) was of the opinion that semi-government or semi-private centers were more effective. Some respondents (41.1%) did not respond to this item by choice.

### Past exposure and initiation of prevention education

A greater majority of students (*n* = 341, 74.1%) reported having previous exposure to drug-prevention education in school as compared to 118 (25.7%) students who did not. As demonstrated in Table 6, the mean age in which students received prevention education was 10.57 years (SD = 5.704), which was younger than the perceived age appropriate for initiating prevention education at 11.68 years (SD = 3.226). A paired-sample *t*-test further indicated that the difference between age of actual exposure and perceived age appropriate for prevention education was significant [*t* (412) = −2.896, *p* < 0.01]. The minimum age in which students were first exposed to drug-prevention education was 6 years. However, the earliest age deemed appropriate to initiate prevention education was 4 years, which is at pre-school level.

**Table 6:** **Descriptive statistics and paired-sample *t*-test comparing differences between age of exposure and perceived age of initiation**.

Variable	Minimum	Maximum	Mean	SD	*t*	df	*p*
Age of exposure (years)	6	20	10.57	5.704	−2.896	412	0.004
Age appropriate for prevention initiation (years)	4	18	11.68	3.226			

### Drug education: Suggestions for improvement

**Figure 2:**
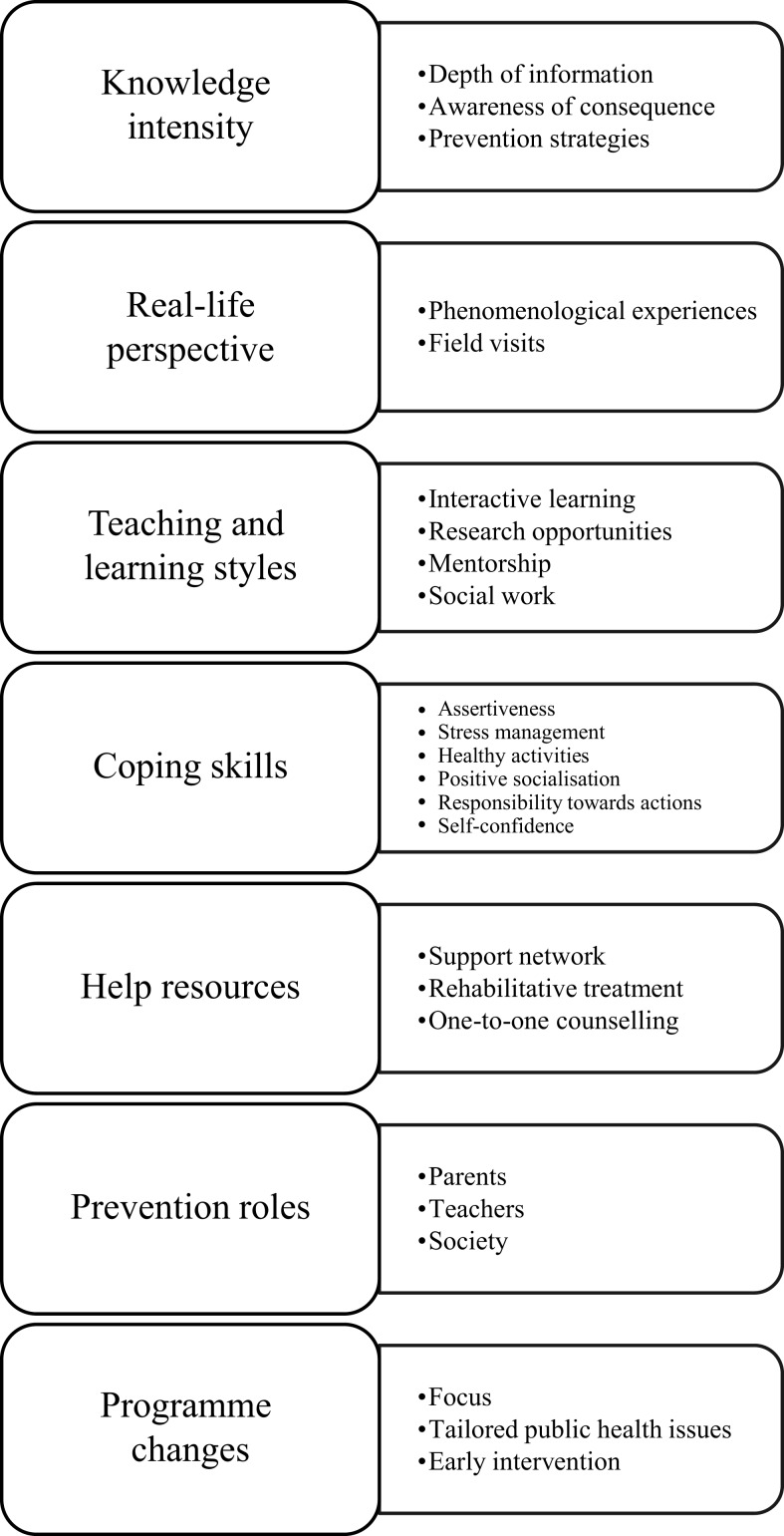
**Thematic summary of suggestions for improvement in drug-prevention education**.

Thematic analysis of students’ open-ended responses revealed seven main themes as shown in Figure 2.

#### Knowledge Intensity

Most responses indicated that in-depth knowledge based on drug abuse causes, consequences, and its effects are required. The students were of the opinion that educating students through subjects such as chemistry, biology, and science would provide them with more exposure toward drug knowledge, how it is produced, and its effect on the body system. This would lead to a deep awareness of the consequences of drug use. The students felt that deeper understanding on the attractiveness of drugs as temporary relief from troubles and a source of fun while socializing versus the complications to one’s health even with recreational use, is required. Moreover, they were interested in having more knowledge about health conditions associated with drug abuse such as AIDS and psychological conditions like bipolar disorders, eating disorders, schizophrenia, and anxiety disorders. The students felt that by truly comprehending the effects of drug use on their health, lifestyle, family, friends, as well as the legal consequences, this would help school children and youths make rational decisions regarding drug use. In-depth information on prevention strategies was also suggested as it could help students be more aware of the various situations in which accidental drug use could occur, help identify the symptoms of addiction, the resources to help friends and family members who are abusing drugs and positive attitudes that should be applied when dealing with drug users.

‘Different kinds of drugs abused daily, how to prevent drug abuse, possible situations that could lead to drug abuse, the dangers of drug abuse.’ [US011]

‘Type of disease that can be caused through drug abuse and how does this type of diseases can be spread.’ [US195]

‘How to spot drugs such as drugs camouflaged as sweets for young children, the behaviours of an addict and withdrawal symptoms, how and where to report especially if it is friends and how to know if they have been drugged …’ [US397]

#### Real-Life Perspective

Field visits and phenomenological experience were also mentioned as important for drug prevention. Students though that by getting a real-life perspective on drug users’ difficulties, the drug use cycle, impacts on families, as well as the physical and mental struggles related to treatment, students would be further encouraged to avoid drug use. They would be motivated to avoid similar experiences for themselves.

‘Do a study trip to a rehabilitation home to let them see in real life, in front of their eyes, the effects of drugs.’ [US032]

‘… students to go to rehab centres or follow on call with the people running the rehab phone lines, who are constantly trying to convince those with drug addiction problem to get help for themselves. No amount of motivational speeches tailored for students would dent the problem except real life first-hand experience.’ [US138]

#### Teaching and Learning Style

Students also suggested learning through research. Some suggested class assignments, which would help increase their awareness and understanding of the effect of drugs on their body. Using interactive learning styles incorporating experiments related to the chemical behavior of various drugs in the body was suggested as a way to increase interest and awareness among students. Messages on drug abuse prevention, it was suggested, could also be presented to students in a creative manner using theater, music performances, and game applications.

Opportunities for mentorship with health professionals involved in rehabilitating drug abuse patients as well as speaking to former drug users to obtain guidance on how to avoid getting involved in drugs was also suggested. This concept could be incorporated through a short-term social work experience during the weekends or school holidays. By helping out at the rehab centers or volunteer in community activities conducted in conjunction with the rehab patients, it was suggested that students would be able to see for themselves how treatment is conducted, the issues faced and what facilities and services are available.

‘More dynamic and interactive activities could have better effects. For example, hands-on workshops (for example, analysing how drugs affect our brain in the lab or something like that). Developing social/cultural programs may also be another good possibility (for example, engaging kids into theatre performance, or music and develop drug prevention themes from their participation in this programs).’ [US054]

‘There should be a mentor and mentee program between and ex-drug user and a pupil to provide guidance and prevent them from getting involved in drugs.’ [US459]

#### Coping Skills

Students made multiple suggestions for coping skills training as well. Teaching stress management using healthy methods such as exercise, meditation, breathing techniques, having a balanced diet, and proper time management was suggested as well as exposure to alternative methods to deal with life stresses, academic pressure, and peer pressure would help them cope more effectively without the need for using performance-enhancing drugs. Students also suggested encouraging healthy lifestyles by introducing a myriad of healthy activities. Simple exercise routines, extra-curricular activities, as well green events could play a part in getting students to incorporate more outdoor activities, learn about indoor games, and activities that would encourage positive socialization as well as how to live healthily without drugs. It was suggested that introducing students to healthy activities and adaptive skills would also lead to positive socialization as being with a group of like-minded peers will encourage them to stay away from negative influences and make better decisions in choosing the right crowd of friends.

At the same time, the students felt that teaching assertiveness skills would encourage them to think independently and logically in resolving their problems rather than being easily influenced by their peers. Assertiveness should go hand-in-hand with instilling self-confidence. Learning to build self-confidence and the belief that the decision they made is the right one after careful deliberation was equally important to the students. Lacking self-confidence will push them into taking the lead from others and result in undesirable actions. Regardless of the consequences of their life decisions, the students felt that early intervention on responsibility toward their own actions would be greatly beneficial to school children and college students.

‘… being confident in yourself to not do the things you don’t want to do rather than following your peers or being influenced by social or external environment.’ [US020]

‘Promote healthy lifestyles and provide strategies to manage life difficulties.’ [US072]

#### Help Resources

The students felt that the scope on rehabilitative treatment information could be widened. Information on available treatment services, treatment approaches, and how to seek help should be publicized so that family members and friends of drug users could provide help where needed. In addition, the students viewed a strong support network with non-judgmental support and advice from health professionals to be important in encouraging young drug users to seek help. The role of family support during and after drug rehab treatment also requires greater emphasis. It was suggested that confidential one-to-one counseling sessions should be made easily available to students so that root problems to stress issues can be resolved. As drugs provide only a temporary escape, facing the issue directly and working it out with a counselor could help find an alternative solution with longer-lasting effects.

‘… programs should also provide information on available local drug rehabilitation for those who have started taking drugs.’ [US017]

‘… emphasis on family support and care for former drug rehab patients after treatment.’ [US235]

‘…one-to-one counselling with certain students. Get to know the root of their problems and make them understand why drugs is bad for them.’ [US401]

#### Prevention Roles

Teachers have an important role in prevention efforts. As the students perceive teachers as role models that primary school children look up to, teachers can educate them about how to solve problems, choosing the right friends, identifying the different types of drugs, and the danger of using drugs. Based on their past experience, the students were aware that some secondary school students tend to assume that they know it all about drugs and may not give their full focus toward health programs conducted by external facilitators. Thus, it was suggested that teachers who have a good rapport with students work closely with this group of students to provide a greater impact. In addition, the organization of open forums and debates on how to prevent drug use and what schools can do to promote drug-free lifestyles, was proposed with the cooperation of parents, teachers, and students.

Besides teachers, parents play a pivotal role in educating their children about drugs. The students generally felt that it would be beneficial to have parental input on how drug abuse cases (i.e., students who were caught using, dealing or exhibiting drug abuse symptoms) should be handled. In addition, clearer guidelines on how parents should explain about drugs were needed as most parents may not be as well-versed on this issue too. Moreover, the students felt that parents need to be more involved in properly monitoring their children’s progress by allocating time to attend counseling sessions between the school counselor and student at certain stages. Similarly, the students felt that the society should have a greater role in drug prevention by ensuring that information on prevention strategies is spread throughout their respective communities. Community and youth leaders should also be well-educated on the topic of drug abuse and be available as a source of advice for troubled members within their community.

‘… citizen’s role to prevent drug abuse.’ [US137]

‘… regular teachers should be the ones educating on drug prevention as they are students’ role models. While external regulators may conduct the programs in school, not many students actually absorb the information. Most secondary school students think they already know enough about drug abuse and prevention, and do not have the heart to focus for sessions of speeches given by facilitators they do not know. Thus, teachers whom students look up to would make the most impact.’ [US175]

‘Drug abuse issues should be discussed more often during assembly, PIBG (Parents and Teachers Association) meeting and during school activities.’ [US267]

#### Program Changes

A change in the program focus was also proposed. The students reported a need for more realistic examples of drug use to be able to relate and empathize with the impact of drug use on the individual’s life. Moreover, prevention activities should be conducted periodically using different approaches to reinforce their knowledge. An area that was often cited for improvement was the public health programs. In particular, it was suggested that programs widen the education scope to include other health and social issues that correlate with drug abuse such as domestic violence and sex education. In addition, the programs should be tailored to address drug use trends such as how to avoid being duped into using drugs unknowingly. Public health activities should also progress with technology and be made available to the public through social media besides the conventional display exhibitions and talks. Reports and articles about drug abuse should be widely published to increase awareness on this topic. Additionally, the students felt that public health programs should be made as part of the early intervention practice. As prevention is often touted as better than cure, students should be educated on drug abuse and situations that they should avoid from an early age, such as not taking food or candy from an unknown individual. Children need to understand the dangers of drugs to the self and how it will worry their parents if they get involved in drug use.

‘… we all know drugs is dangerous, however curiosity of the sensation of getting high might lead people to try it. the immediate side effects of drugs won’t surface until much later. So I think it’s important to actually get to see other people’s experience in drugs, something we can imagine and emphasise with.’ [US095]

‘… it is fundamental for them to know it earlier. Prevention is better than cure.’ [US119]

‘Conduct tailored programs and activities that will teach youths how to prevent themselves from accidently getting involved in drugs.’ [US334]

## Discussion

The overall objective of this study was to investigate the perceptions of university students related to drug use and availability as well as drug abuse and treatment services. In particular, this study was interested in getting non-user opinions on drug abuse to determine what areas might require more attention when implementing drug education and prevention programs.

The findings demonstrated that there were both differences and similarities in students’ perceptions of commonly used substances compared to a 5-year analysis by NADA (7). While ecstasy and cannabis were cited as most commonly used and easily available by students, NADA (7) cited opiates as the most commonly abused substance in Malaysia from 2009 to 2013 with 45% of their subjects using heroin or morphine. Although heroin was also ranked by students as most commonly used and in the second tier of easily available substances, morphine was mid-rank in both commonality and availability and opium received a rank of 7 in commonality and availability. According to the NADA statistics, the use of cannabis and amphetamine-type substances (ATS) (i.e., ecstasy, methamphetamines, and amphetamines) are consistently in the third to sixth place rankings across 5 years while synthetic drugs such as ketamine and nimetazepam have decreased drastically in use from the year 2011 to 2013 (6, 7, 9). This was similar to the current finding, in which methamphetamines were ranked third in terms of commonality but obtained a rank of 6 in availability. While ketamine has been popular in party circles since the 1980s, nimetazepam is another designer substance that has been increasingly popular since entering the market in 2003. Nimetazepam is also known as Erimin, and is a benzodiazepine derivative, which possesses sedative, hypnotic, and anxiolytic properties. This substance is usually found in the form of 5 mg tablets and is therefore, also prevalently known among drug users as Erimin-5. Originally, it was prescribed to patients suffering from severe insomnia but is commonly misused by Malaysian drug users as an alternative to opiates and amphetamines (22). Psychoactive pills and ketamine were perceived as the most difficult substances to obtain, with a ranking of 10. In addition, ketamine was also ranked as the least commonly used substance, together with ketum leaves.

Although there were differences in perception, this does not reflect that students have inaccurate perceptions in any way. Instead, their perceptions could reflect the actual situation within their surroundings (i.e., college, university, workplace, and neighborhood) as they are expected to answer the SPQ based on existing knowledge, observation, or experience. As the statistics by NADA were based on substance use profiles obtained from drug users who were admitted for rehabilitative treatment, there should be caution in using this data for interpreting the actual availability and commonality of substances. There is a possibility that the substance types that are accounted for in the reports may be biased since drug users who were admitted into treatment are often heavy or long-term users, and may have dabbled in a variety of substances including “hard” drugs. This research has subsequently highlighted another limitation to previous research, which is a lack of information about the availability and commonality of substances within the community. While findings from past research on drug user populations such as Vento et al. (23) and Bersani et al. (24) have generally found that drug users view ecstasy or MDMA and psychoactive pills as chemically similar in effect, the current study found an interesting viewpoint in non-user perceptions. Additional qualitative feedback from the pilot study indicated that the students viewed these substances differently in its context of use. The students perceived ecstasy or MDMA as a recreational drug because it was more often associated with its role as party drugs, while psychoactive pills were viewed as substances that were used to treat psychological disorders.

Findings on factors contributing to drug abuse demonstrated that female students generally rated personal sufferings, problems with family, coping with life, curiosity, associations with drug-dependent peers, and influence of media portrayals as important reasons for drug use. However, only unemployment was rated significantly higher by female students in comparison to male students. Male students rated “not educated” and “is weak-willed” higher than females although this difference was not significant. The view of unemployment as a cause of substance abuse is supported by Henkel (25), whose comprehensive review concluded that unemployment plays a dual role as a significant risk factor and an outcome of drug use. The perceptions of our Malaysian sample partially coincided with those reported by Cirakoglu and Isin (21), who found in a Turkish sample that females tend to attribute drug use to problem coping. However, in their study, males tended to attribute drug use to sensation seeking (i.e., questionnaire items related to curiosity), which was not found in this study.

Responses on relapse factors showed that students perceived lack of family support as a major reason for relapse, followed by lack of self-efficacy and peer influence in importance. Research from the United States generally concurs with these perceptions, providing evidence that loss of support from family as well as encountering peers still involved in drugs are significant risk factors for relapse (26). Ibrahim et al. (27) also found a strong relationship between low self-efficacy and relapse tendencies. Patients who are released from treatment with inadequate preparation for re-integrating into society, such as lacking the necessary job and self-sufficiency skills, are more likely to experience low self-efficacy and a resultant higher risk of falling back into substance abuse.

Looking at gender differences in perceptions of effective strategies for preventing drug relapse, female students gave higher scores to four strategies: “building supportive social networks,” “learning stress management,” “maintaining constant communication with recovery doctors,” and “consulting rehabilitation centers.” These were in contrast to the findings by Cirakoglu and Isin (21), whose Turkish female participants were less likely to view help-seeking, self-change, and social activity as methods of overcoming drug relapse. The only strategy, which the male students rated higher than female students, was “forgetting the past and make life changes,” although this difference was not significant. Cirakoglu and Isin (21), by contrast, found that males were more likely to view avoidance as instrumental in preventing relapse.

The students in this sample preferred using internet websites and blogs as their primary resource to search for information, followed by newspaper and magazine articles, as well as brochures, pamphlets, or posters. This finding was similar to a study by Stetina et al. (28) on 9268 respondents from North America, Australia, as well as English-speaking and German-speaking European countries. It was interesting to note that social sites and books were the least preferred resource for university students. This can be attributed to the reason that it is difficult to identify the reliability and credibility of information in social media, since rumors and misinformation can be spread quickly. The need for quick information at their fingertips, however, is making books less popular. Moreover, books on substance abuse are often scarce in libraries and are costly to buy. Findings such as these further emphasize the important role of the Internet as a resource for information on substance types and drug abuse patterns. With this in mind, Schifano and colleagues (29) created a web tool called the Psychonaut 2002 Project. The objective of this project was to provide professionals from the field of substance abuse with updated and reliable information on drug scenarios within European countries. Nevertheless, it is equally important that this information be disseminated to the public, as they are the target population who requires awareness about new designer substances and the danger of illicit substances being sold on the net as herbal supplements, such as marijuana and ecstasy (30).

Interestingly, social media and internet websites were preferred methods for sharing information, as they are able to reach out to selected or wide circles of audience with a simple click of the mouse. Radio and television were also perceived as good educational resources due to their wide use and availability. However, our findings indicated that a large number of individuals did not seek out information on their own, so the role of prevention education should not be overlooked as a way of reaching students and young adults who do not actively search for and exchange such information.

The analysis on knowledge about rehabilitative services and the differences in treatment effectiveness demonstrated that a major proportion of students perceived that they have knowledge about the drug rehab programs that are available in Malaysia. A slightly higher proportion of students also believed that there is a difference in treatment effectiveness between government and private rehab centers with 48.9% of students viewing private centers as more effective than government rehab centers. The general perception of private rehab being more effective than public rehab services may stem from age-old beliefs that private healthcare is more efficient and responsive to patients’ needs (31). As private rehab and healthcare are often associated with large funds to provide advanced treatment, engage experts and provide ample space within treatment facilities without a long wait list (32), this has further fueled their beliefs despite high treatment costs (33).

Precisely, 74.1% of students in this sample reported having past exposure to drug-prevention education in school. It was interesting to note that the recommended age for initiating prevention education was slightly older (*M* = 11.68) than the mean age in which students received prevention education (*M* = 10.57). Although efforts were made by NADA to introduce drug education early since pre-school through the TUNAS program (4), the views of students on when it should be initiated should also be taken in full consideration. For instance, it may be more appropriate to conduct drug education between the ages of 11 and 12 years as their formal operational stage of cognitive development begins. It is around this age that children begin to show great increases in their ability to think abstractly, to plan logically, and to understand the long-term consequences of their actions (34). It is also a time when children begin seriously considering their identity and how they would like to fit into society as a whole.

Qualitative feedback from students about drug education and prevention programs revealed that there is much improvement needed in the existing programs. Besides increasing the depth and scope of drug information, the students preferred the use of interactive teaching and learning methods rather than talks and exhibitions, which have the tendency to be rather dry. In addition, the students were also interested in having realistic examples through experience-sharing or an opportunity to do volunteer work at rehab centers. Besides drug prevention, drug education should be tailored to address medical conditions and social issues that are related to drug abuse. Drug prevention would be more effective with access to help resources such as help lines, the contacts of health professionals and the dissemination of coping skills. Parents, teachers, and society need to play a more active role in drug prevention and develop prompt procedures to detect, identify, and seek treatment for underage children and youths who abuse drugs.

There are several points within this study that requires further discussion. This study utilized purposive sampling to recruit participants although this sampling method is synonymously associated with qualitative research. Purposive sampling is a non-probability method, which views sampling as a strategic series of choice about whom, where, and how the study is done (35). Decisions were made by the researchers in regards to the participants, which are to be included in the sample based on a variety of research criteria such as specialist knowledge or unique perspective of the research issue or the capacity and willingness to participate (35). Thus, the method of sampling needs to be tied to the research objective and the working context of the research. Purposive sampling was chosen because this study had specific objectives in examining the perceptions of drug abuse issues among university students who have not engaged in drug use. This was tied with the researchers’ aim in understanding non-user views about drug abuse and what factors could potentially place this sample group at risk for drug use, at the present moment or in the near future. Thus, participants who fulfilled the recruitment criteria were sought, as they would be able to contribute appropriate data, both in terms of relevance and depth. Although the data from this study, coming from five different states in both Peninsular and East Malaysia, comprise a wide geographical representation of Malaysia, the study has several limitations. First, as with all self-report-based research is the possibility of response bias. The SPQ is a self-report measure, thus there is a probability that some students may have responded in a way that would reflect well on them, despite the fact that the SPQ is administered anonymously. Second, students may be providing their opinions on drug types without a good deal of knowledge, as this was not a test of knowledge, *per se*, but essentially an opinion survey. There was a possibility that some students lacked familiarity with the legal names of substances, and studies such as Corazza et al. (36), proposed that adolescents and young adults may be more familiar with substances marketed using attractive street names or associated with popular brand names. Nevertheless, the drug types were listed according to their legal names in the SPQ due to the reason that a majority of students in Malaysia do have awareness about the legal names of illicit substances, as they have been exposed to this knowledge through drug-prevention programs in school. In addition, the numerous commercial or street names for each substance, which consequently varies based on location and country, could equally posed an issue in which students may have been exposed to different street or brand names, depending on the media, which they were frequently exposed to (i.e., Western or Eastern films, TV shows, social media sites, Internet sites). Third, the students were mostly sampled from higher education institutions in urban areas. There could be slight differences in relation to drug types that are commonly used and easily accessible in rural areas. Future research could further expand on this area by including students and working professionals from both rural and urban areas to gain a more comprehensive understanding of non-user perceptions across the population of Malaysia. Furthermore, this study has a higher proportion of responses from female students as compared to males. It could be argued that males may have greater awareness about drug abuse issues since a higher proportion of drug users are males and were more likely to have experimented with new psychoactive substances and used recreational drugs, as compared to female drug users (37). However, this does highlight the question of whether females really lack awareness about new psychoactive substance. There is a possibility that females may choose not to use particular substances despite their awareness about it, as they are motivated by different reasons for drug use. Several studies have demonstrated that males were more likely to use substances for experimentation as compared to females (38), whose drug use was more highly associated with mood and anxiety disorders (39), eating disorders (40), as well as severe premenstrual syndrome or premenstrual dysphoric disorder (41). Although the findings related to gender differences may not be representative of the academic population, gender differences in perception, related to reasons for drug use and relapse prevention strategies should be taken seriously in developing gender-sensitive drug prevention. Future studies should consider increasing the sample size and width of participant demographics, in addition to examining how diverse beliefs, culture, and prior exposure to drug information in the media could influence perceptions about drug abuse.

This research has important implications toward the evaluation of drug education and prevention programs. Understanding the perceptions of non-user populations is essential toward identifying misconceptions and knowledge gaps about illicit drugs, effective prevention strategies, and factors of drug abuse and relapse so that remedial steps can be taken to prevent them from experimenting or misusing drugs. Getting public feedback on drug-prevention programs and including their opinions into future planning is the way forward in efforts to obtain active participation from school children, young adults, parents, teachers, and the community. The qualitative responses on how current drug education and prevention programs could be improved in particular suggest that an active community role is increasingly important toward effective prevention. Prevention activities in school and higher education institutions only are no longer sufficient, and students would like to see greater cooperation and contributions across the community. Media outlets, for example, can ensure that appropriate and accurate educational messages are adequately circulated. Schools, colleges, and universities should continue conducting drug education programs in an on-going manner, and parents should be involved with their child’s life, providing support, communication, and a healthy environment as well as working together with teachers to identify any warning signs if they arise (42). In response to the students’ calls for greater community involvement, influential action groups should be formed to discuss and initiate changes in the national drug use policy (42). Training the community to identify specific drugs and underlying issues that could lead to drug use will encourage greater monitoring of young children and adolescents who at-risk. The community can also play a greater role in drug prevention by providing feedback in assessments to evaluate the effectiveness of prevention programs to weed out ineffective strategies, and focus time and effort on those who do work.

It was unsurprising to find that the students in this sample perceived that treatment facilities and services provided by private rehabilitation centers were more effective. These beliefs about public and private rehabilitative services have long existed. This suggests that more work should be done to improve the quality of services and facilities in public treatment centers to gain the public’s confidence. Dissemination of help information during drug-prevention activities was an aspect that required more emphasis to students. This gap could be fulfilled by increasing public access to available treatment, counseling, and support services offered by NADA and various NGOs such as PEMADAM and PENGASIH. Additionally, findings on the students’ preferred medium for learning and sharing will help target suitable media for disseminating drug information and prevention strategies. Responses on contributory factors and relapse prevention strategies do show the influence of gender on the attribution of triggers for drug misuse and drug relapse prevention. However, more research is needed to understand gender roles on drug use perceptions and translating its influence into tailoring effective drug-prevention messages. The overall data collection of student and public perceptions on drug abuse has significant implications toward the creation of learning materials to educate the community as a whole. An example would be the creation of an online educational database to provide the public with updated information about new substance types that they should be wary of, latest research findings, who and where to seek help for drug users, coping skill modules as well as guidelines on how to identify drug use. This blog is currently being conceptualized and developed by the researchers.

To conclude, this research was able to shed some light on students’ opinions in regards to their drug education and prevention; preferred media for learning and sharing information; insights on drug knowledge and prevention strategies; and perceptions about treatment services. In the future, it is hoped that such feedback from non-user populations can be factored into drug prevention and other public health programs.

## Conflict of Interest Statement

The authors declare that the research was conducted in the absence of any commercial or financial relationships that could be construed as a potential conflict of interest.

## References

[1] Scorzelli JF. Has Malaysia’s drug rehabilitation effort been effective? Int J Psychosoc Rehabil (2009) 13(2):21–4.1324990

[2] Yuen MK Zahid: stamp out drug addiction The Star [Internet]. Nation. (2013). Available from: http://www.thestar.com.my/News/Nation/2013/06/21/Zahid-Stamp-out-drug-addiction.aspx

[3] Malaysian Psychiatric Association. Drug Addiction [Internet]. Kuala Lumpur: Malaysian Psychiatric Association (2006) [cited 2006 Jul 6]. Available from: http://www.psychiatry-malaysia.org/article.php?aid=90.

[4] Harun HN Gazali E. New approach for drug treatment and rehabilitation News Straits Times [Internet]. Nation. (2013) [cited 2013 July 2]. Available from: http://www.nst.com.my/latest/new-approach-for-drug-treatment-and-rehabilitation-1.311198

[5] Priya SS Centre’s treatment helps drug addicts move on The Star [Internet]. Community. (2013) [cited 2013 Aug 20]. Available from: http://www.thestar.com.my/News/Community/2013/08/20/Centres-treatment-helps-drug-addicts-move-on.aspx

[6] National Anti-Drug Agency (NADA) (Malaysia). Laporan dadah tahun [Internet]. Kajang: Ministry of Home Affairs, Policy, Planning and Research Department (2012) [cited 2012 Dec]. Available from: http://www.adk.gov.my/html/laporandadah/2012/Laporan%20Dadah%20Dis%202012.pdf

[7] National Anti-Drug Agency (NADA) (Malaysia). Laporan dadah bulan Disember [Internet]. Kajang: Ministry of Home Affairs, Policy, Planning and Research Department (2013) [cited 2013 Dec]. Available from: http://www.adk.gov.my/html/laporandadah/2013/Laporan%20Dadah%20Bulan%20Disember%202013.pdf

[8] National Anti-Drug Agency (NADA) (Malaysia). Laporan dadah Disember [Internet]. Kajang: Ministry of Home Affairs, Policy, Planning and Research Department (2010) [cited 2010 Dec]. Available from: http://www.adk.gov.my/html/laporandadah/Disember%202010.pdf

[9] National Anti-Drug Agency (NADA) (Malaysia). Laporan dadah bulan Disember [Internet]. Kajang: Ministry of Home Affairs, Policy, Planning and Research Department (2011) [cited 2011 Dec]. Available from: http://www.adk.gov.my/html/laporandadah/2011/Laporan%20Dadah%20Bulan%20Disember%202011.pdf

[10] Priya SS These days, addicts aren’t just homeless runaways. The Star [Internet]. Community (2013) [cited 2013 Aug 20]. Available from: http://www.thestar.com.my/News/Community/2013/08/20/A-change-in-profile-These-days-addicts-arent-just-homeless-runaways.aspx

[11] Caldeira KM Kasperski SJ Sharma E Vincent KB O’ Grady KE Wish ED College students rarely seek help despite serious substance use problems. J Subst Abuse Treat (2009) 37(4):368–78. 10.1016/j.jsat.2009.04.005 19553064 PMC2783958

[12] Getz L Treating professionals with substance use disorders. Social Work Today (2012) 12(6):14.

[13] United Nations Office on Drugs and Crime. Drug Statistics and Trends. Vienna: United Nations (2010) [cited 2014 Oct 20]. Available from: http://www.unodc.org/documents/wdr/WDR_2010/2.0_Drug_statistics_and_Trends.pdf

[14] Mazibuko F. Drugs and young people: prevention and therapeutic models of intervention within the context of social development Proceedings of the 29th ICSW International Conference on Social Welfare; 2000 Oct 23-27; Cape Town, South Africa. Available from: http://www.icsw.org/global-conferences/mazibuko.htm

[15] Shewan D Dalgarno P. Evidence for controlled heroin use? Low levels of negative health and social outcomes among non-treatment heroin users in Glasgow (Scotland). Br J Health Psychol (2005) 10(t1):33–48. 10.1348/135910704X14582 15826332

[16] Ersche KD Jones PS Williams GB Smith DG Bullmore ET Robbins TW. Distinctive personality traits and neural correlates associated with stimulant drug use versus familial risk of stimulant dependence. Biol Psychiatry (2013) 74(2):137–44. 10.1016/j.biopsych.2012.11.016 23273722 PMC3705207

[17] Sanchez ZM Oliveira LG Nappo SA. Main reasons for non-use of illicit drugs by young population exposed to risk situations. Rev Saúde Pública (2005) 39(4):599–605. 10.1590/S0034-89102005000400013 16113910

[18] Bejarano J Ahumada G Sanchez G Cadenas N de Marco M Hynes M Perception of risk and drug use: an exploratory analysis of explanatory factors in six Latin American countries. J Int Drug Alcohol Tob Res (2011) 1(1): 9–17.

[19] Matthew-Simmons F Love S Ritter A. DPMP Monograph Series (Monograph No. 17): a review of Australian public opinion surveys on illicit drugs. Sydney, NSW: National Drug and Alcohol Research Centre (2008).

[20] Larimer M Kilmer J Lee C College student drug prevention: a review of individually-oriented prevention strategies. J Drug Issues (2005) 35:431–56 10.1177/002204260503500210

[21] Cirakoglu OC Isin G. Perception of drug addiction among Turkish university students: causes, cures and attitudes. Addict Behav (2005) 30:1–8. 10.1016/j.addbeh.2004.04.003 15561444

[22] Devaney M Reid G Baldwin S Situational analysis of illicit drug issues and responses in the Asia-Pacific region ANCD Research Paper 12. Canberra, ACT: Australian National Council on Drugs (2005).

[23] Vento AE Martinotti G Cinosi E Lupi M Acciavatti T Carrus D Substance use in the club scene of Rome: a pilot study. Biomed Res Int (2014) 2014:6. 10.1155/2014/617546 25243163 PMC4163412

[24] Bersani FS Corazza O Albano G Valeriani G Santacroce R Posocco FBM 25C-NBOMe: preliminary data on pharmacology, psychoactive effects, and toxicity of a new potent and dangerous hallucinogenic drug. Biomed Res Int (2014) 2014:6. 10.1155/2014/734749 25105138 PMC4106087

[25] Henkel D. Unemployment and substance use: a review of the literature (1990-2010). Curr Drug Abuse Rev (2011) 4:4–27. 10.2174/1874473711104010004 21466502

[26] National Institute on Drug Abuse. Are relapse risk factors different in offender populations? How Should Drug Abuse Treatment Deal With These Risk Factors? [Internet] (2014) [cited 2014 Dec 21]. Available from: http://www.drugabuse.gov/publications/principles-drug-abuse-treatment-criminal-justice-populations/are-relapse-risk-factors-different-in-offender-popu

[27] Ibrahim F Kumar N Samah AB Self efficacy and relapsed addiction tendency: an empirical study. Soc Sci (2011) 6(4):277–82 10.3923/sscience.2011.277.282

[28] Stetina BU Jagsch R Schramel C Maman TL Kryspin-Exner I. Exploring hidden populations: recreational drug users. Cyberpsychology (2008) 2(1).Available from: http://cyberpsychology.eu/view.php?cisloclanku=2008060201&article=117109206

[29] Schifano F Leoni M Martinotti G Rawaf S Rovetto F. Importance of cyberspace for the assessment of the drug abuse market: preliminary results from the Psychonaut 2002 project. Cyberpsychol Behav (2003) 6(4):405–10. 10.1089/109493103322278790 14511453

[30] Forman RF Marlowe DB McLellan AT. The internet as a source of drugs of abuse. Curr Psychiatry Rep (2006) 8:377–82. 10.1007/s11920-006-0039-6 16968618

[31] Rosenthal G Newbrander W. Public policy and private sector provision of health services. Int J Health Plann Manage (1996) 11(3):203–16. 10.1002/(SICI)1099-1751(199607)11:3<203::AID-HPM432>3.0.CO;2-0 10162428

[32] De Costa A Diwan V. ‘Where is the public health sector?’ Public and private sector healthcare provision in Madhya Pradesh, India. Health Policy (2007) 84(2–3):269–76. 10.1016/j.healthpol.2007.04.004 17540472

[33] Babar ZD Izham MI. Effect of privatization of the drug distribution system on drug prices in Malaysia. Public Health (2009) 123(8):523–33. 10.1016/j.puhe.2009.06.011 19665741

[34] McLeod SA Formal Operational Stage (2010) [cited 2013 Aug 20]. Available from: http://www.simplypsychology.org/formal-operational.html

[35] Oliver P Purposive sampling. In Victor J, editor. The SAGE Dictionary of Social Research Methods [Internet]. SAGE Publications Ltd (2006) [cited 2015 Mar 7]. Available from: http://dx.doi.org/10.4135/9780857020116

[36] Corazza O Valeriani G Bersani FS Corkery J Martinotti G Bersani G “Spice,” “Kryptonite,” “Black Mamba”: an overview of brand names and marketing strategies of novel psychoactive substances on the web. J Psychoactive Drugs (2014) 46(4):287–94. 10.1080/02791072.2014.944291 25188698

[37] Corazza O Simonata P Corkery J Trincas G Schifano F. “Legal highs”: safe and legal “heavens”? A study on the diffusion, knowledge and risk awareness of novel psychoactive drugs among students in the UK. Riv Psichiatr (2014) 49(2):89–94. 10.1708/1461.16147 24770577

[38] McCabe SE Cranford JA Boyd CJ Teter CJ. Motives, diversion and routes of administration associated with nonmedical use of prescription opioids. Addict Behav (2007) 32(3):562–75. 10.1016/j.addbeh.2006.05.022 16843611 PMC1766373

[39] Conway KP Compton W Stinson FS Grant BF. Lifetime comorbidity of DSM-IV mood and anxiety disorders and specific drug use disorders: results from the National Epidemiologic Survey on Alcohol and Related Conditions. J Clin Psychiatry (2006) 67(2):247–57. 10.4088/JCP.v67n0211 16566620

[40] Hudson JI Hiripi E Pope HG Jr Kessler RC. The prevalence and correlates of eating disorders in the National Comorbidity Survey Replication. Biol Psychiatry (2007) 61(3):348–58. 10.1016/j.biopsych.2006.03.040 16815322 PMC1892232

[41] Terner JM de Wit H. Menstrual cycle phase and responses to drugs of abuse in humans. Drug Alcohol Depend (2006) 84(1):1–13. 10.1016/j.drugalcdep.2005.12.007 16413143

[42] Pentz MA Preventing drug abuse through the community: multicomponent programs make the difference. Proceedings of the National Conference on Drug Abuse Prevention Research: Presentations, Papers and Recommendations; (1996) Sep 19–20; Washington, DC. Bethesda, MD: National Institute on Drug Abuse Available from: http://archives.drugabuse.gov/meetings/CODA/Community.html

